# Supplementary material for: Lipid‐induced lysosomal damage after demyelination corrupts microglia protective function in lysosomal storage disorders
Source: EMBO J. 2018 Dec 7;38(2):e99553. doi: 10.15252/embj.201899553 (PMC6331723; doi:10.15252/embj.201899553)
Supplement: Supplementary file 1 — Appendix [file EMBJ-38-e99553-s001.pdf]

Gabandé-Rodríguez et al.

Lipid induced lysosomal damage after demyelination corrupts microglia protective function in Lysosomal Storage Disorders

-Appendix-

Table of contents:

Appendix Legends to Figures S1-S11

Appendix Figures S1-S11

APPENDIX SUPPLEMENTARY LEGENDS TO FIGURES

Figure S1. Oligodendrocyte number is not altered by ASM deficiency or PLX treatment.

(A) Immunofluorescence staining against Olig2 (green) in the Cb of ASMko and wt mice treated or not with PLX for 2 months. DAPI staining shows cell nuclei. Scale bar, 100 μm.

(B) Mean ± SEM number of Olig2 positive cells per area in the different mouse groups (*n* = 7 mice per group).

Figure S2. Impaired axonal myelination in ASMko mice.

(A) Immunofluorescence staining against the myelin marker MBP (green) and the axonal marker NF200 (red) in the Cb of ASMko and wt mice treated or not with PLX for 2 months (first column) and its 3D rendered replicates (second column). Scale bars, 20 μm. The images evidence the reduced amount of myelin wrapping NF200 positive axons in ASMko and ASMko PLX mice compared to wt and wt PLX mice. Magnified images of the selected areas are shown in the third column and as 3D rendered replicates in the fourth column. Scale bars, 10 μm.

(B) Mean ± SEM percentage of demyelinated axons (*n* = 7 mice per group). *P**<0.05; *P****<0.001.

Figure S3. Myelin debris phagocytosis by Arg-1 positive microglia of ASMko mice.

Immunofluorescence staining against MBP (green), Arg-1 (red) and Iba-1 (yellow). Arrowheads show Arg-1 positive microglia-containing MBP aggregates indicative of Myelin debris. Scale bar, 20 μm.

Figure S4. Arg-1 positive microglia accumulates lipofuscin deposits.

Immunofluorescence staining against Arg-1 (red) and autofluorescence from lipofuscin like deposits (green) in the Cb of ASMko mice. Arrowheads indicate Arg-1 positive microglia containing lipofuscin deposits. In the lower panel lipofuscin autofluorescence was quenched by Sudan Black (SB) treatment. Scale bar, 30 μm.

Figure S5. LMP in microglia cultured from postnatal ASMko mice.

(A) Lysenin staining in postnatal primary microglia from wt and ASMko mice. Scale bar, 20 µm.

(B) Mean ± SEM lysenin intensity (as % of control) (*n* = 3 independent cultures, Student´s ***t***-test).

(C) Lysotracker-Red staining in postnatal primary microglia from wt and ASMko mice. Scale bar, 10 µm.

(D) Western blot of CathB levels (for both the precursor (pro-CathB) and the cleaved-mature forms) in the culture media from ASMko and wt postnatal primary microglia. Staining of Ponceau-S is shown as loading control.

(E) Mean ± SEM percentage increase in pro-CathB and CathB levels in the culture media from wt and ASMko postnatal microglia (*n* = 3 independent cultures, Student´s ***t***-test).

Data information: *P**<0.05.

Figure S6. TMEM119 expression is reduced in F4/80 positive cells of the cerebellum in ASMko mice.

(A) Immunofluorescence staining against TMEM119 (green) and F4/80 (red) in the cerebellum of wt and ASMko mice showing TMEM119 positive (arrowheads) and negative/low expressing (asterisks) cells. Scale bar, 50 µm.

(B) Mean ± SEM intensity of TMEM119 in F4/80 positive cells per area in wt and ASMko mice (*n* = 6 mice per group). *P***<0.005.

Figure S7. Inhibition of the P2X7 receptor does not avoid CathB secretion in BMDMs from ASMko and wt mice.

(A) Western blot of CathB levels (for both the precursor (pro-CathB) and the cleaved-mature forms) in the culture media from ASMko and wt BMDMs treated or not with the P2X7 inhibitor A740003. Ponceau-S staining is shown as a loading control.

(B) Mean ± SEM of CathB levels in the culture media from ASMko and wt BMDMs treated or not with A740003 (*n* = 5 independent cultures, Student´s ***t***-test). *P**<0.05; *P***<0.005.

Figure S8. CathB positive microglia in the brain of a NPA patient.

(A) Immunohistochemistry staining against CathB in the brain of NPA affected and control 3-year old children. Arrowheads indicate microglia showing increased and diffuse CathB staining in the NPA patient. Nuclei are stained with hematoxilin. Scale bar, 10 μm.

(B) Immunofluorescence staining against Iba-1 and CathB in the Cb of a NPA patient. Scale bar, 50 μm (left). Magnified image of the selected area showing co-localisation of Cath B and Iba-1 positive cells. Scale bar, 20 μm (right).

Figure S9. Microglia death is not increased in the Cb of ASMko and wt mice.

(A) Immunofluorescence staining against cleaved Caspase 3 (red). DAPI staining shows cell nuclei. Arrowheads indicate apoptotic cells, positive for cleaved Caspase 3. Scale bar, 50 μm.

(B) Immunofluorescence staining against TUNEL (red). DAPI staining shows cell nuclei. Arrowheads indicate apoptotic cells, positive for TUNEL. Scale bar, 50 μm.

(C) Immunofluorescence staining against cleaved Caspase 3 (red) colocalizing with F4/80 (green) positive (up) and negative (down) cells in the cb of a ASMko mouse. DAPI staining shows cell nuclei. Scale bar, 10 μm.

(D) Immunofluorescence staining against TUNEL (red) colocalizing with F4/80 (green) positive (up) and negative (down) cells in the cb of a ASMko mouse DAPI staining shows cell nuclei. Scale bar, 10 μm.

(E) Mean ± SEM of total number of cells (left) or of F4/80 positive cells (right) positive for cleaved Caspase 3 per mm^2 in the cb of wt and ASMko (n = 5 wt mice and 11 ASMko mice, Mann–Whitney ***U*** - test).

(F) Mean ± SEM of total number of cells (left) or of F4/80 positive cells (right) that were TUNEL positive per mm^2 in the cb of wt and ASMko mice (n = 10 mice per group, Mann–Whitney ***U***-test).

Data information: P*<0.05; P**<0.005.

Figure S10. Interleukin 1b levels are reduced in the Cb of ASMko mice.

Milliplex analysis of IL-1b levels in cerebellar extracts from wt and ASMko mice (n = 5 mice per group).

Figure S11. Ponceau S staining.

(A) Ponceau S staining corresponding to the Western blot shown in Figure 5A.

(B) Ponceau S staining corresponding to the Western blot shown in Figure 5O.

A

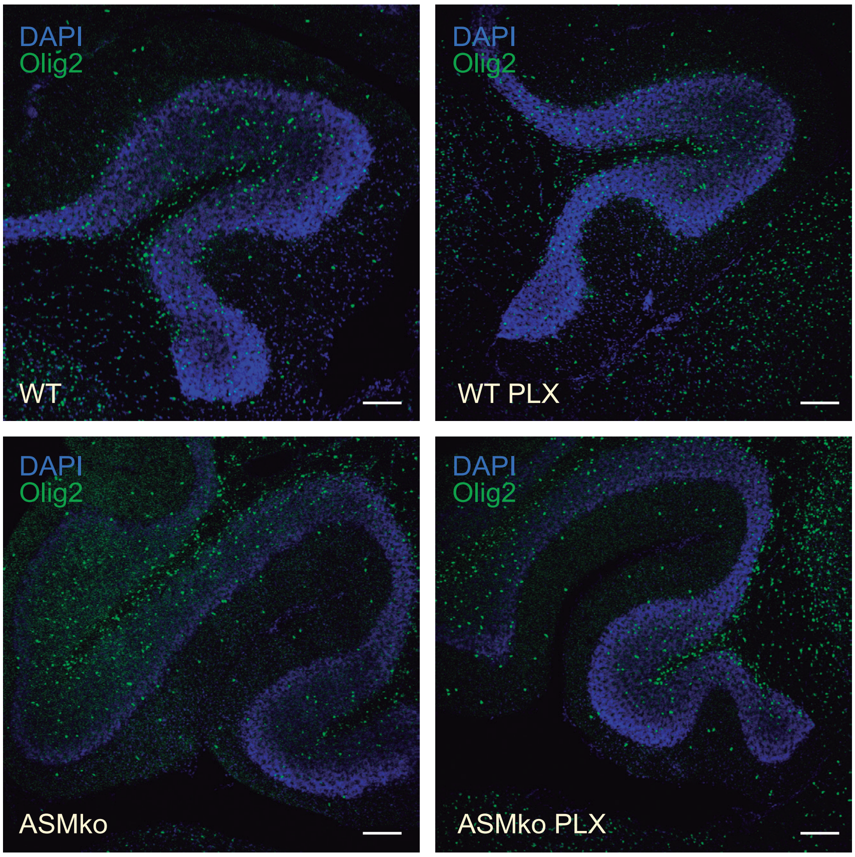

B

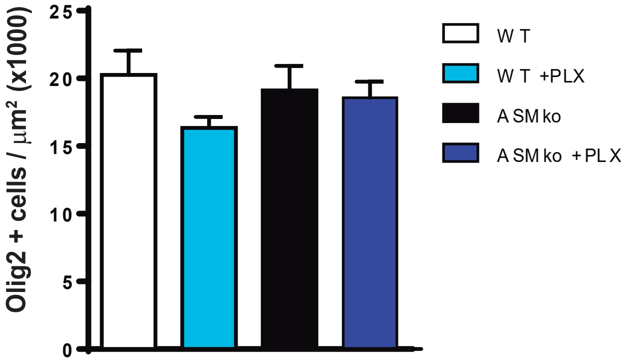

Appendix Fig.S1

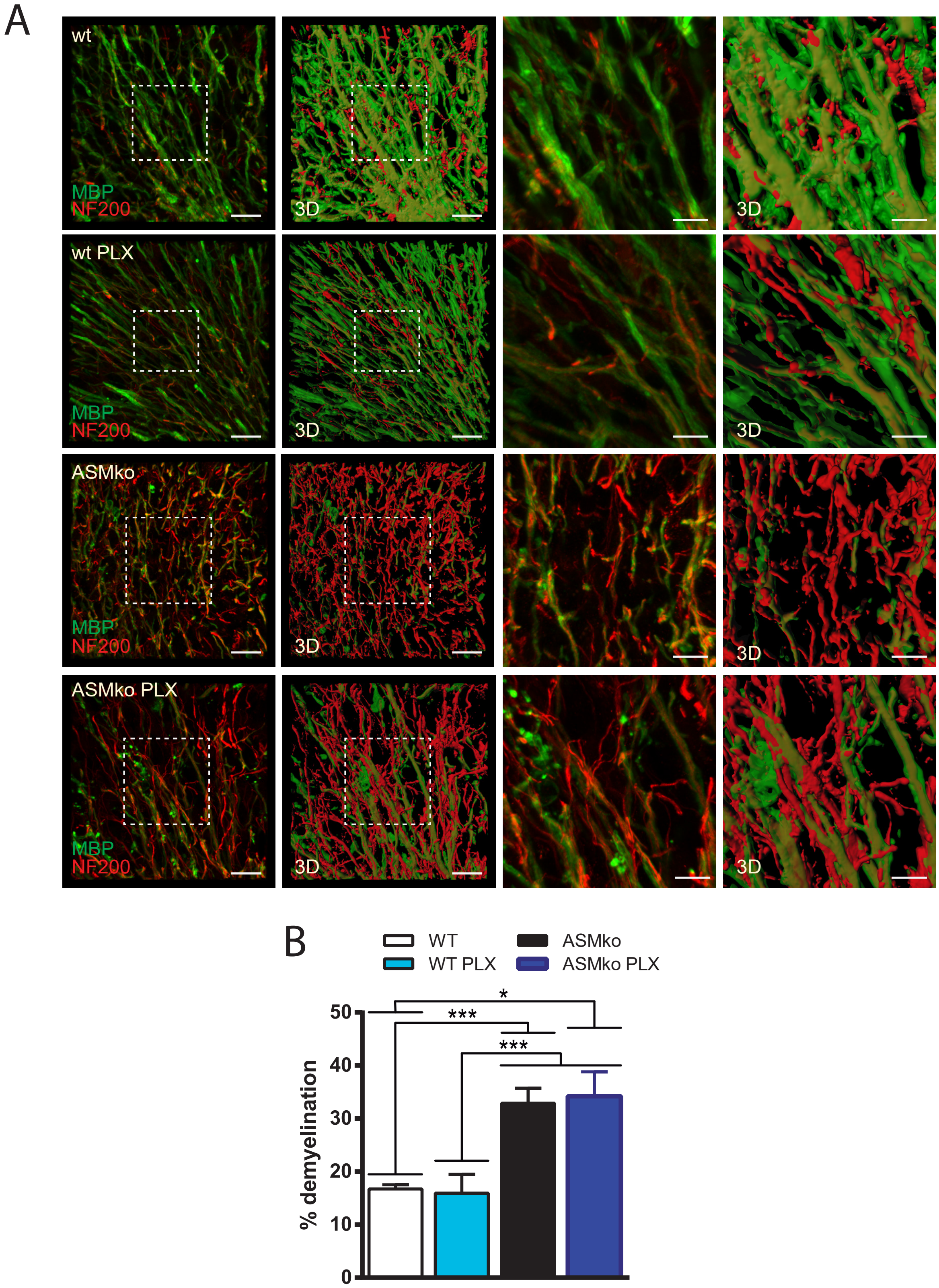

Appendix Fig.S2

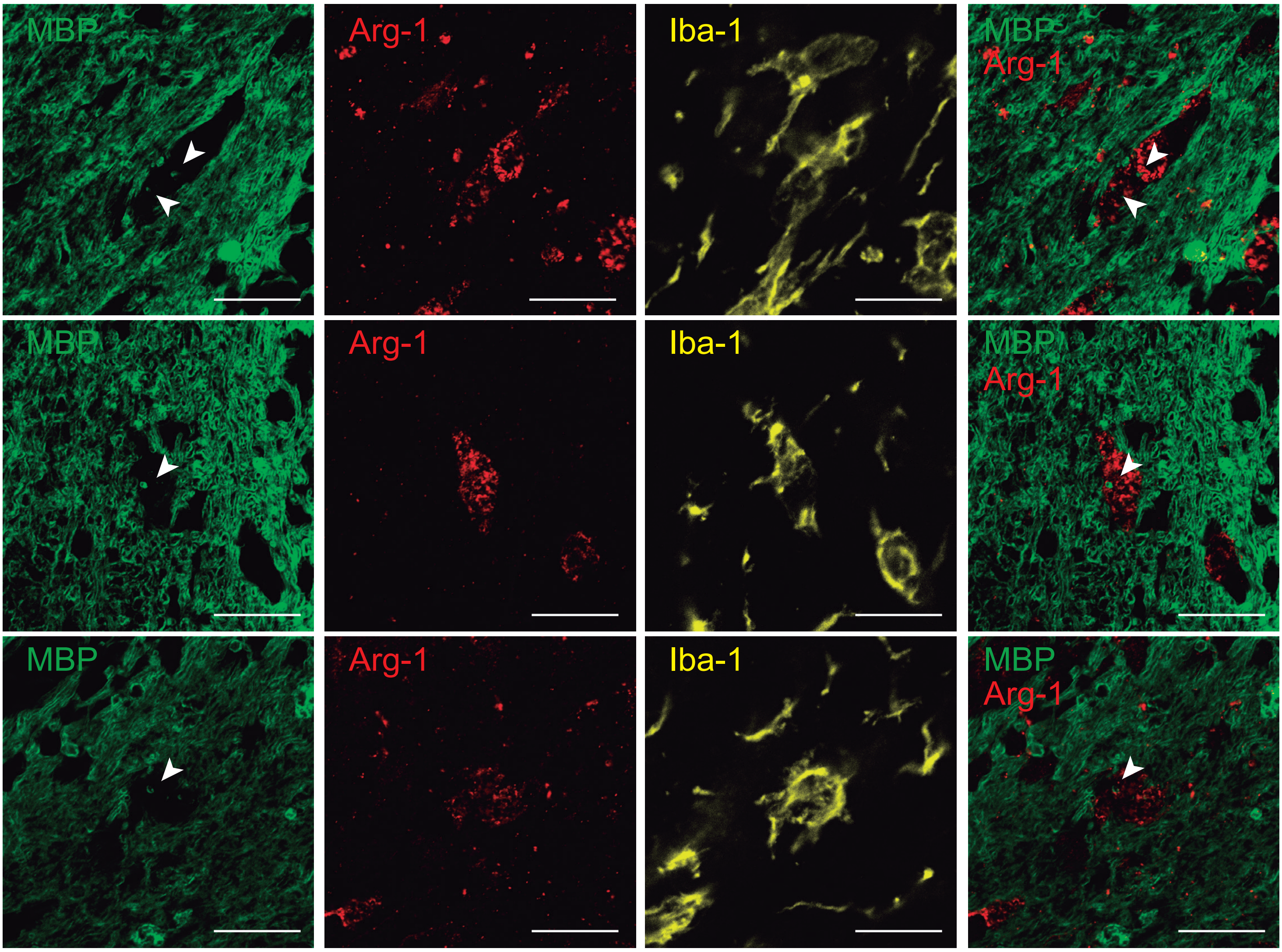

Appendix Fig.S3

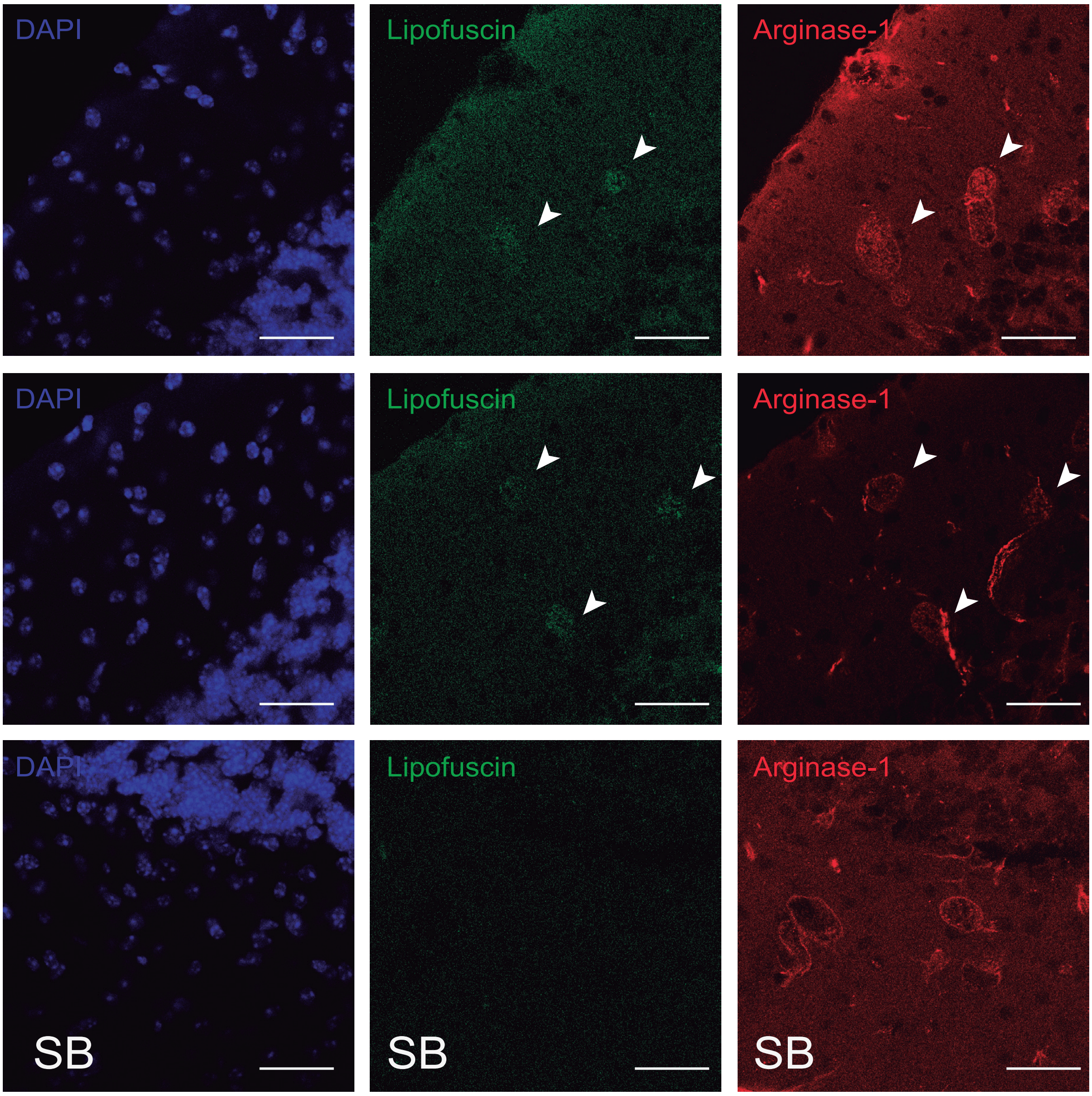

Appendix Fig.S4

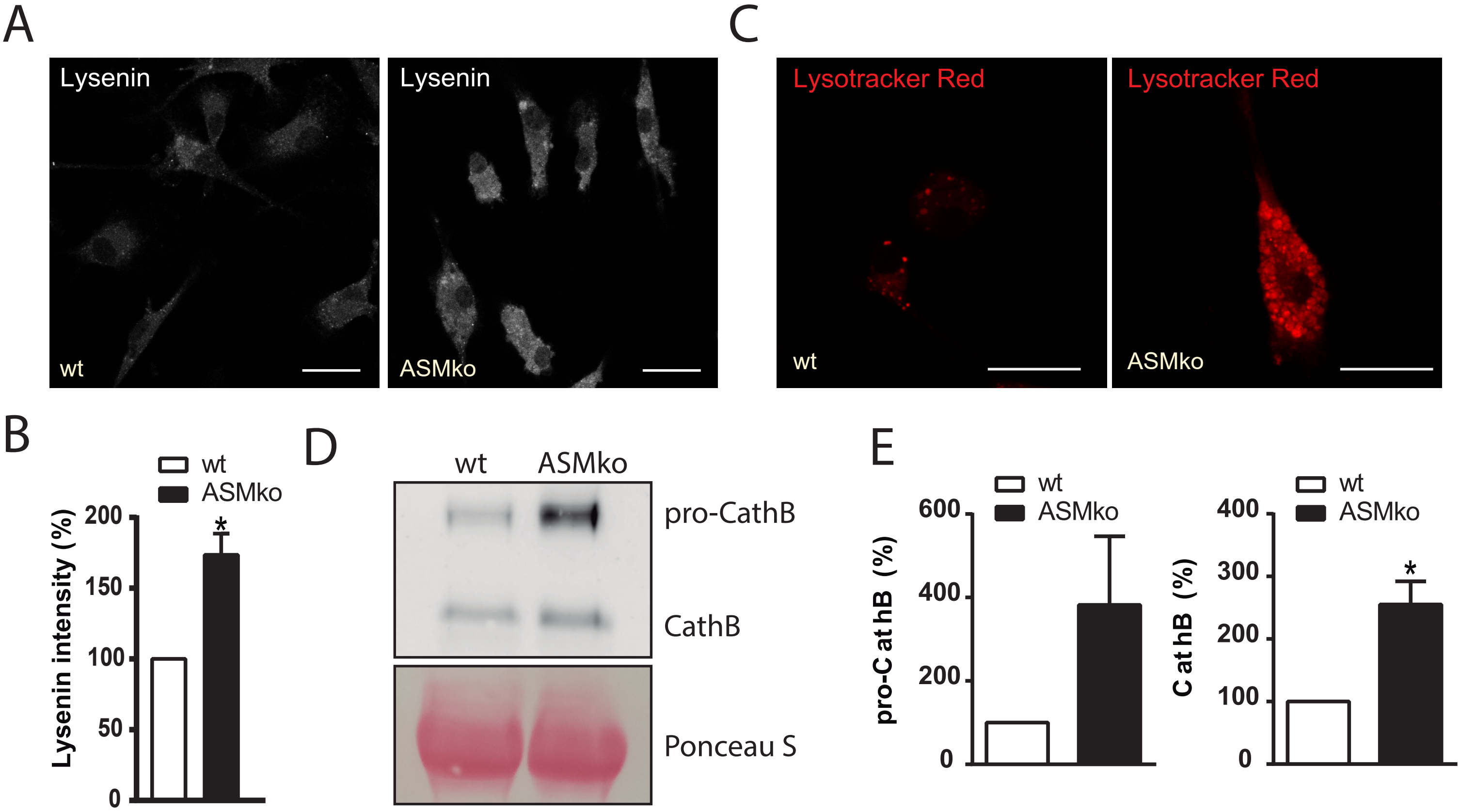

Appendix Fig.S5

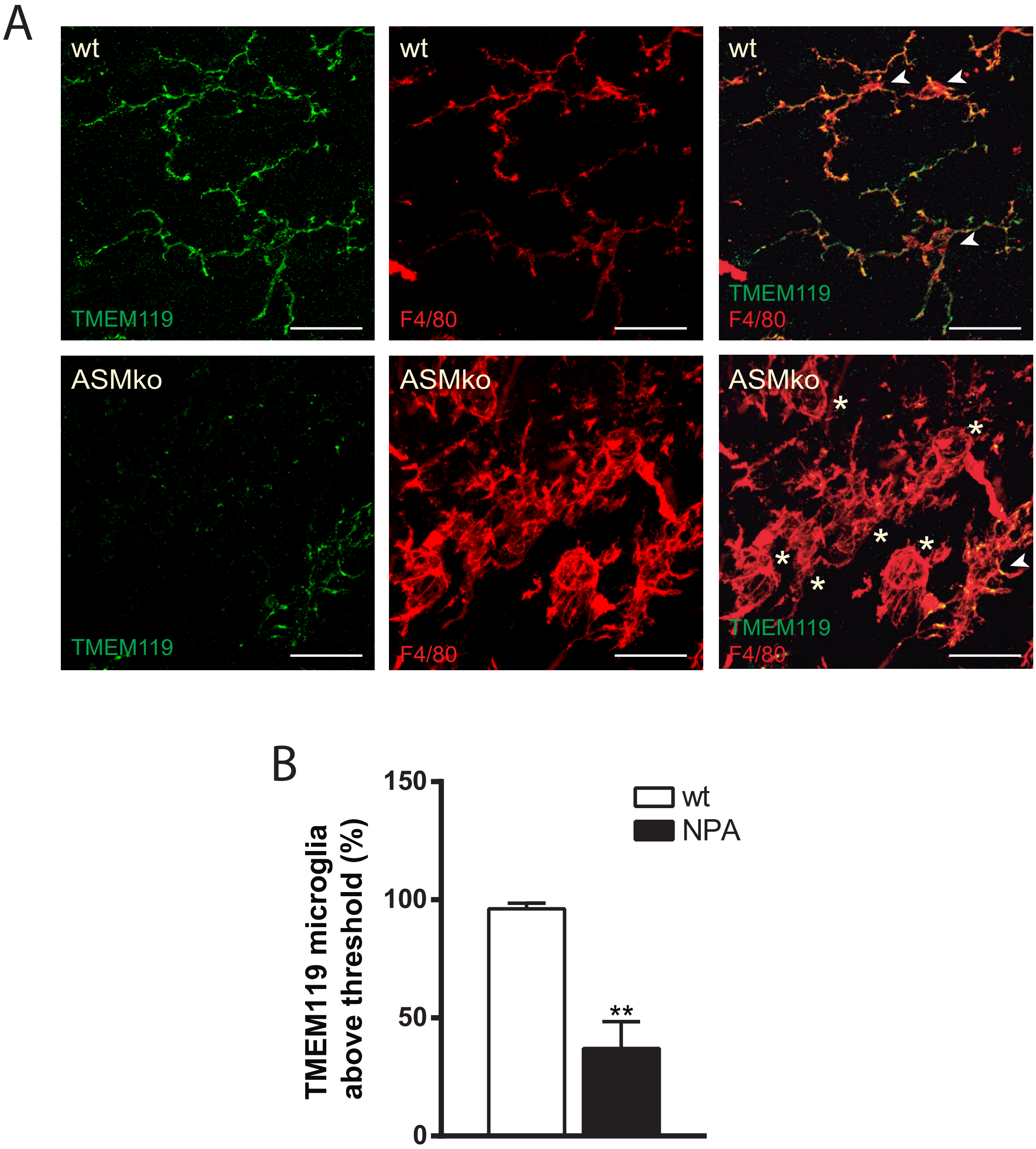

Appendix Fig.S6

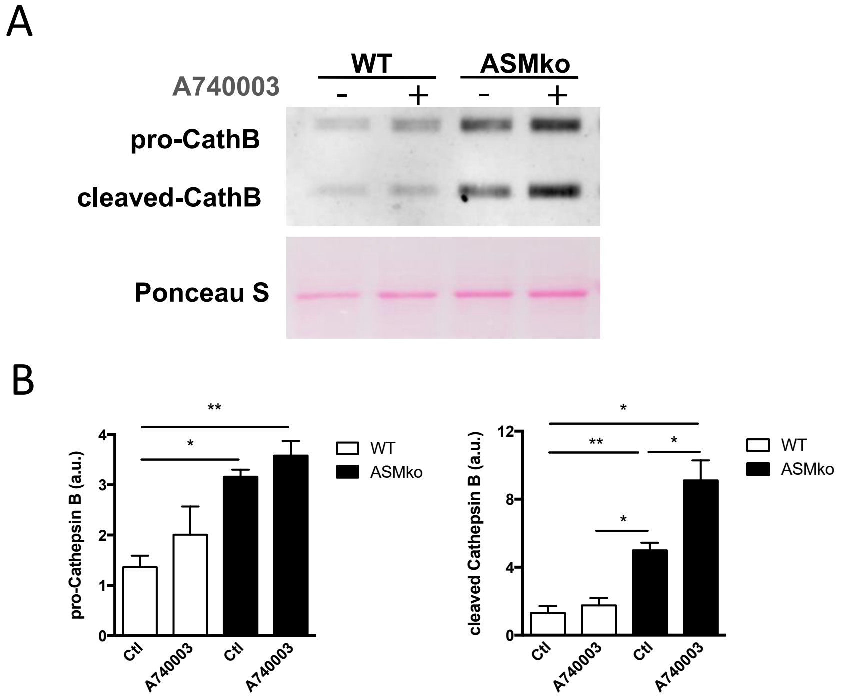

Appendix Fig.S7

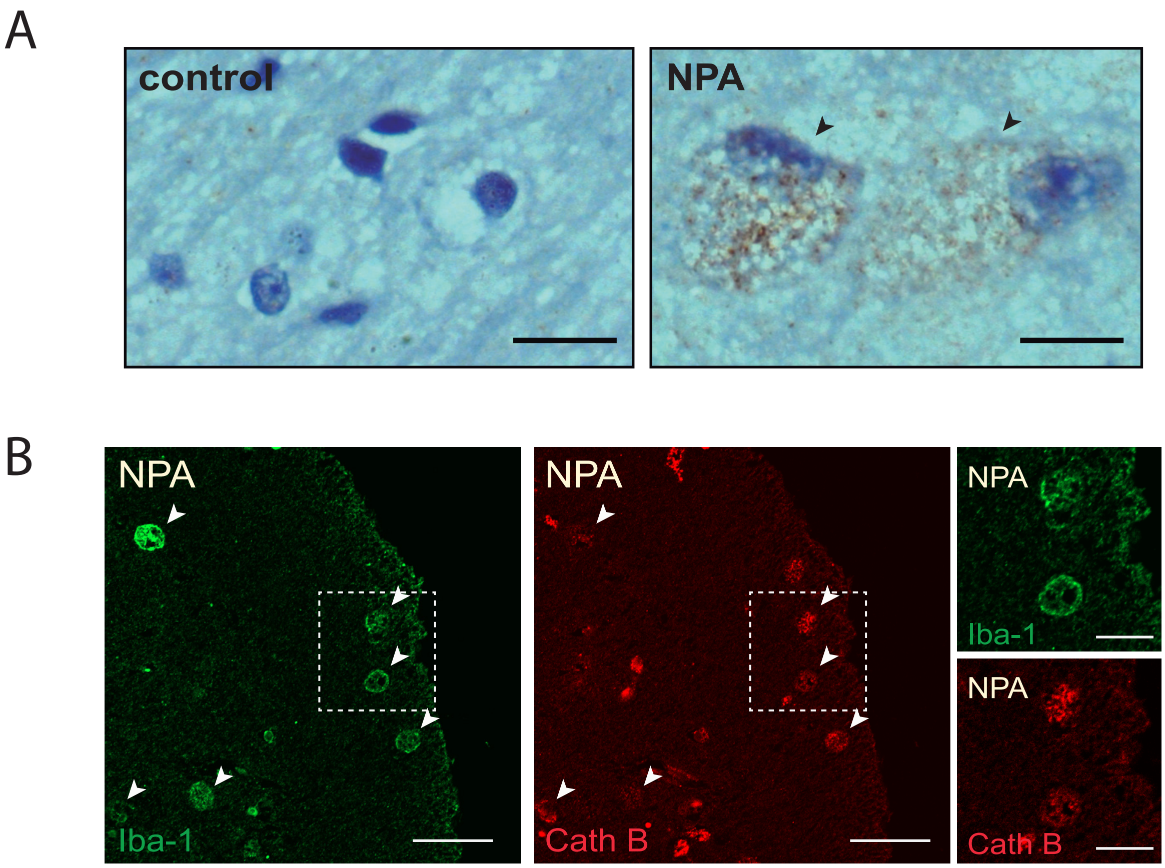

Appendix Fig.S8

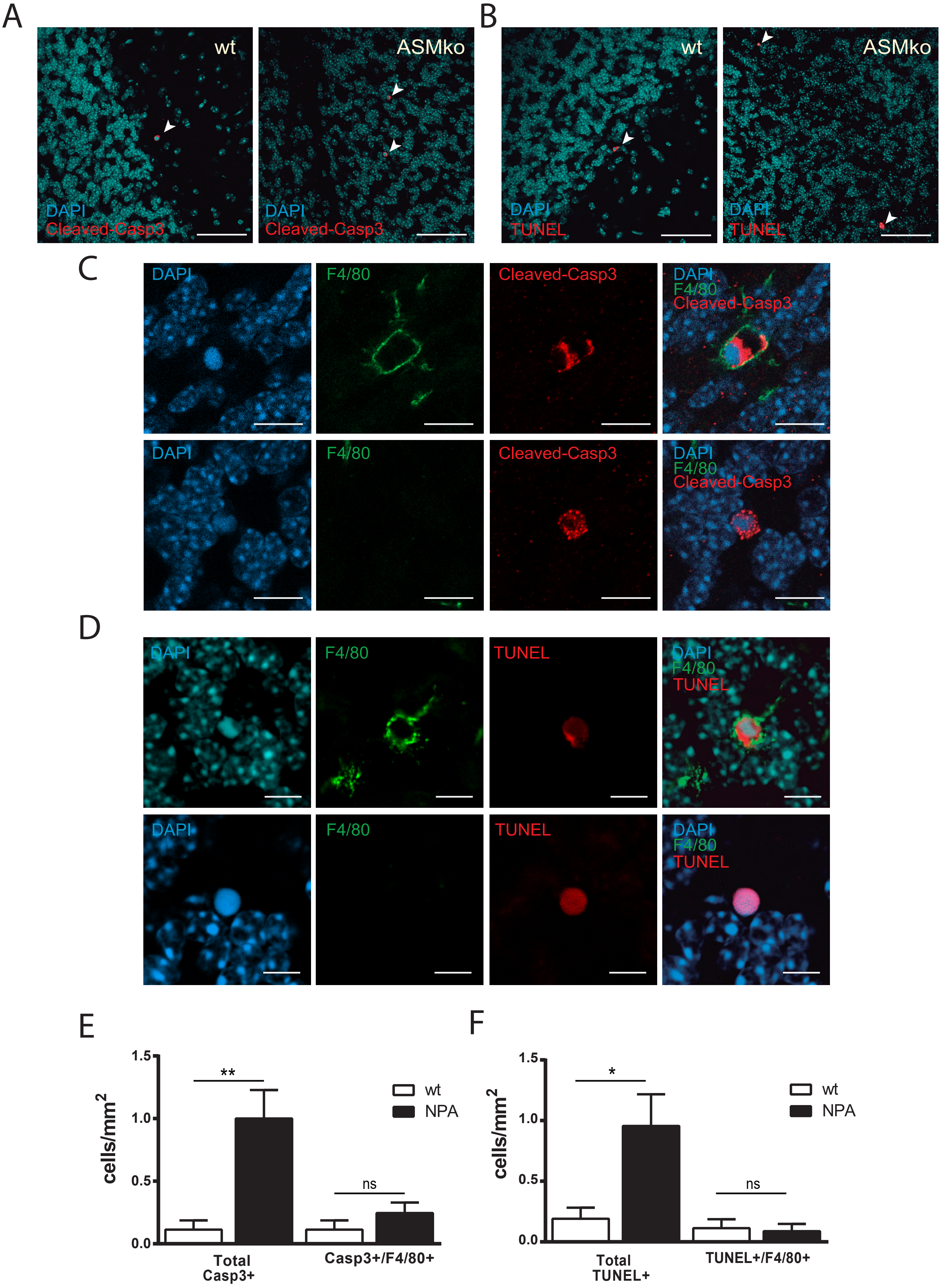

Appendix Fig.S9

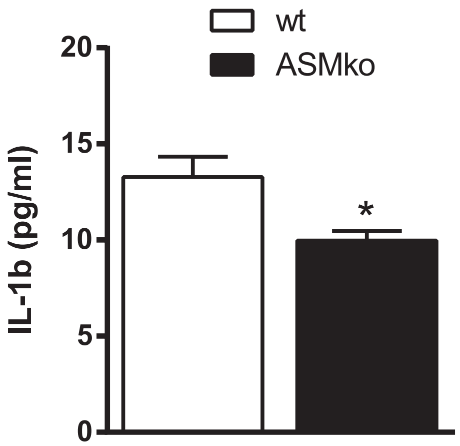

Appendix Fig.S10

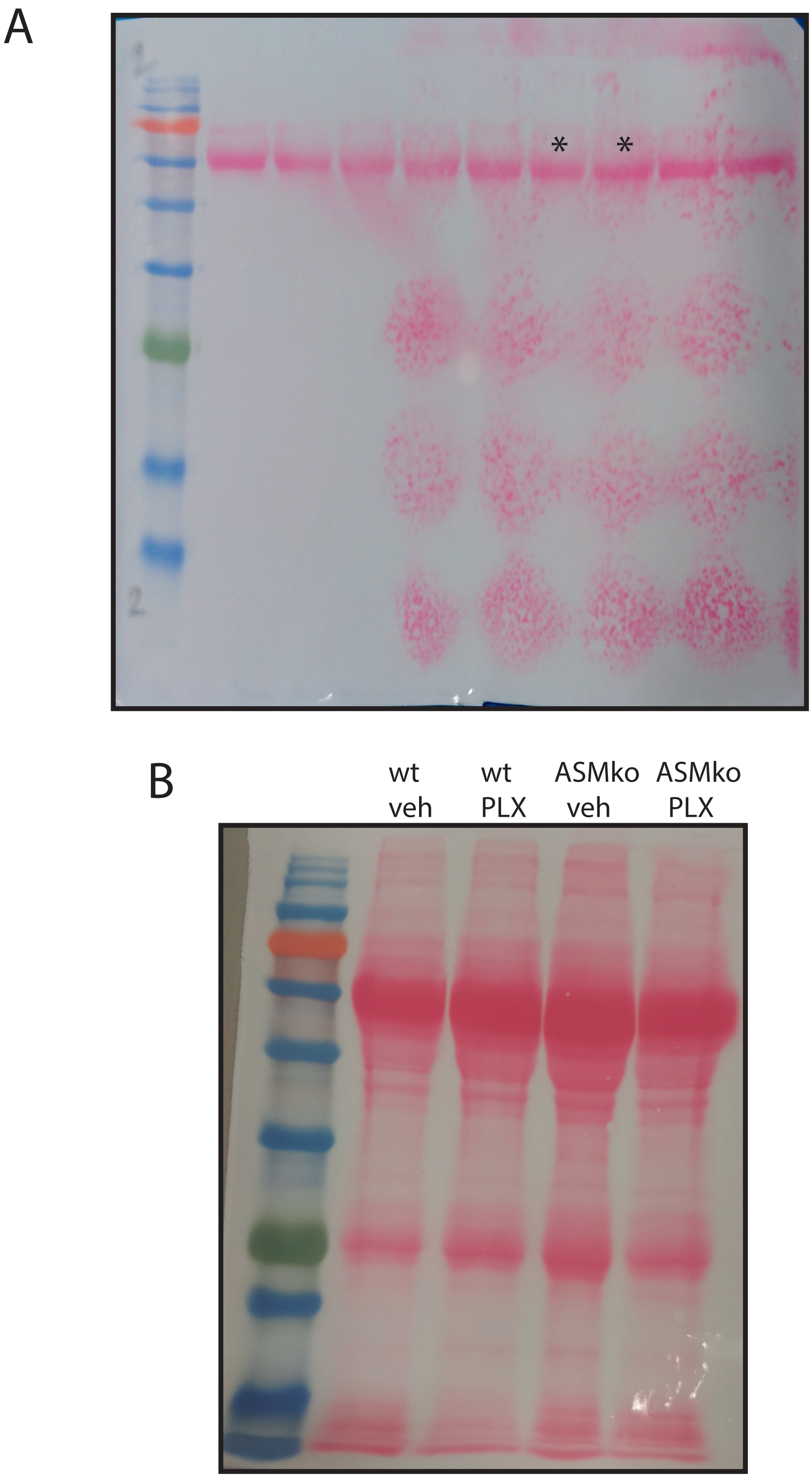

Appendix Fig.S11
