# Supplementary figures and images for: Lipid‐induced lysosomal damage after demyelination corrupts microglia protective function in lysosomal storage disorders
Source: EMBO J. 2018 Dec 7;38(2):e99553. doi: 10.15252/embj.201899553 (PMC6331723; doi:10.15252/embj.201899553)

Appendix Fig. S5 D

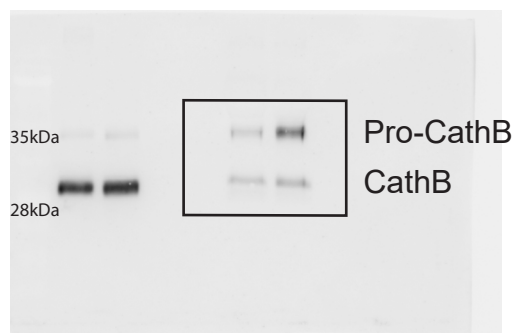

Supplement: Supplementary file 2 — Source Data for Appendix [file EMBJ-38-e99553-s004.zip › Appendix_Fig_S5_SD.pdf]

Appendix Fig. S7A

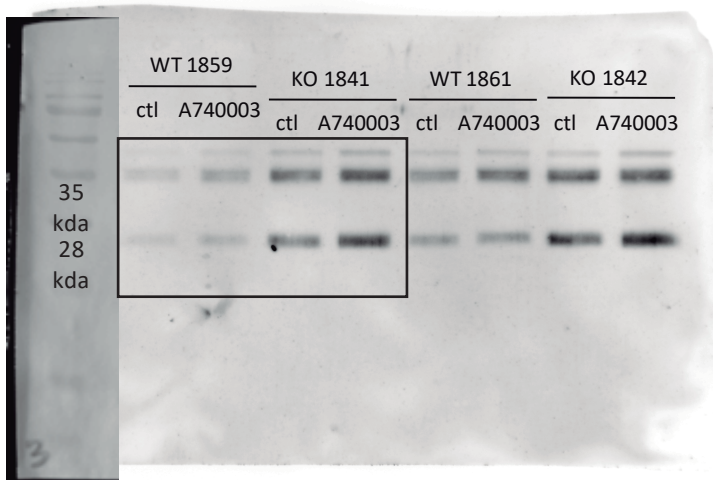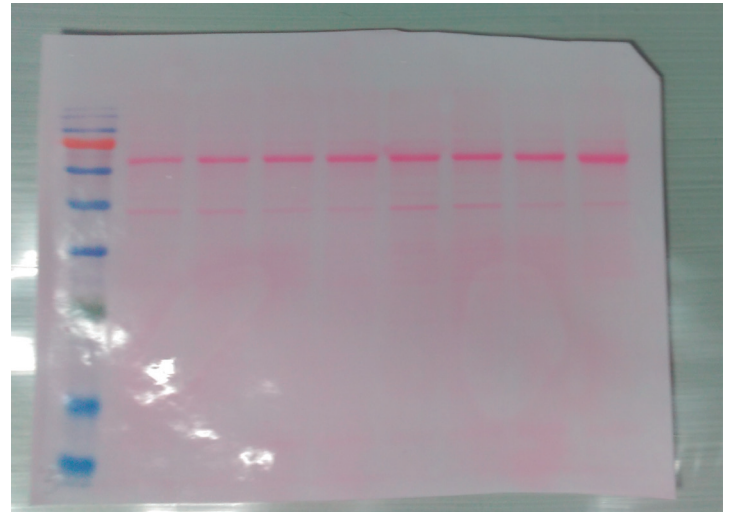

Supplement: Supplementary file 2 — Source Data for Appendix [file EMBJ-38-e99553-s004.zip › Appendix_Fig_S7_SD.pdf]

Fig. 4 F

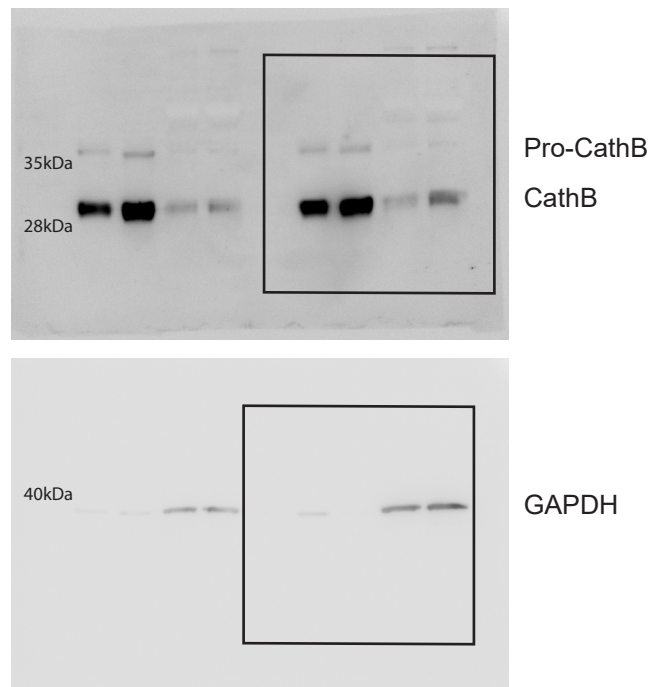

Supplement: Supplementary file 4 — Source Data for Figure 4 [file EMBJ-38-e99553-s002.pdf]

Fig. 5 A

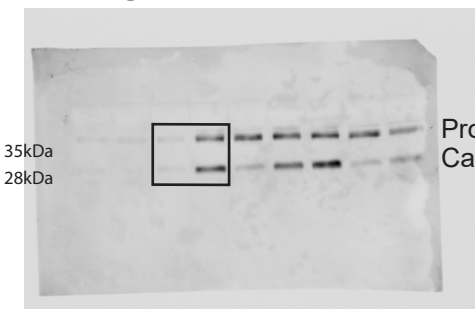

Fig. 5 G

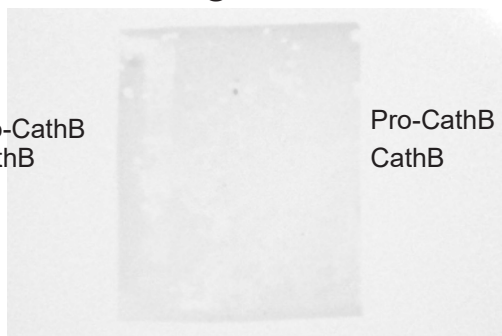

Fig. 5 H

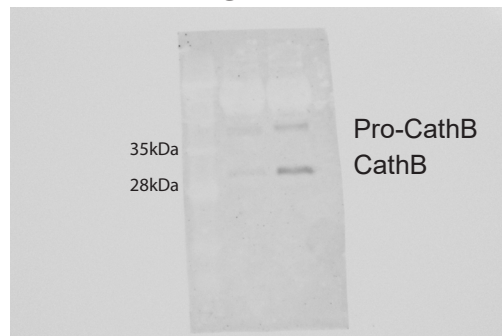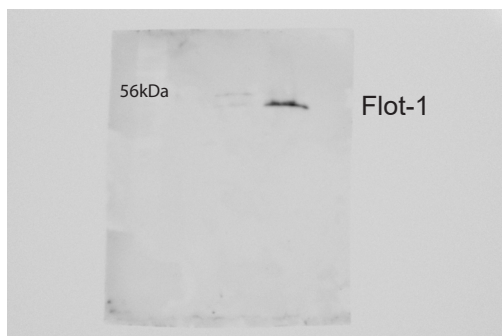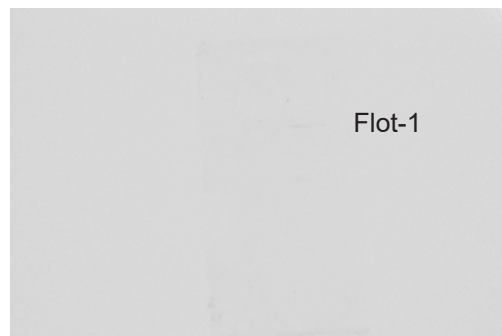

Fig. 5 L

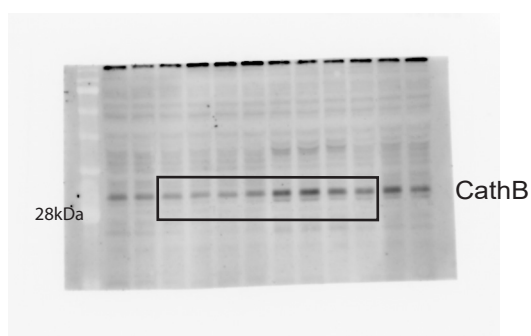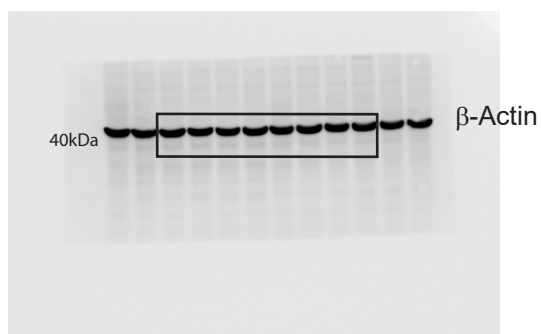

Fig. 5 O

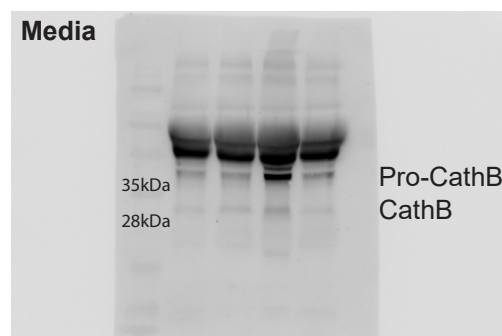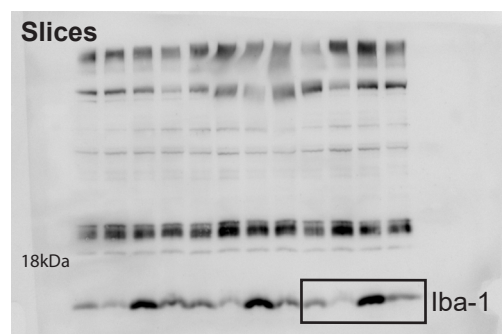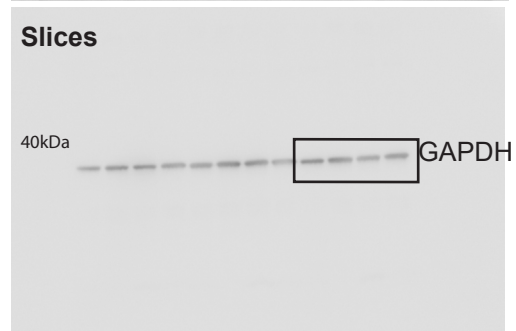

Supplement: Supplementary file 5 — Source Data for Figure 5 [file EMBJ-38-e99553-s003.pdf]
